# Randomized comparison of single dose of recombinant human IL-12 versus placebo for restoration of hematopoiesis and improved survival in rhesus monkeys exposed to lethal radiation

**DOI:** 10.1186/1756-8722-7-31

**Published:** 2014-04-06

**Authors:** Zoya Gluzman-Poltorak, Sarita R Mendonca, Vladimir Vainstein, Hue Kha, Lena A Basile

**Affiliations:** 1 Neumedicines Inc., 133 North Altadena Drive, Suite 310, 91107 Pasadena, CA, USA

**Keywords:** IL-12, HSARS, Hematopoiesis, Total body irradiation, Syndrome, Radiation

## Abstract

**Background:**

The hematopoietic syndrome of the acute radiation syndrome (HSARS) is a life-threatening condition in humans exposed to total body irradiation (TBI); no drugs are approved for treating this condition. Recombinant human interleukin-12 (rHuIL-12) is being developed for HSARS mitigation under the FDA Animal Rule, where efficacy is proven in an appropriate animal model and safety is demonstrated in humans.

**Methods:**

In this blinded study, rhesus monkeys (9 animals/sex/dose group) were randomized to receive a single subcutaneous injection of placebo (group 1) or rHuIL-12 at doses of 50, 100, 250, or 500 ng/kg (groups 2–5, respectively), without antibiotics, fluids or blood transfusions, 24–25 hours after TBI (700 cGy).

**Results:**

Survival rates at Day 60 were 11%, 33%, 39%, 39%, and 50% for groups 1–5, respectively (log rank p < 0.05 for each dose vs. control). rHuIL-12 also significantly reduced the incidences of severe neutropenia, severe thrombocytopenia, and sepsis (positive hemoculture). Additionally, bone marrow regeneration following TBI was significantly greater in monkeys treated with rHuIL-12 than in controls.

**Conclusions:**

Data from this study demonstrate that a single injection of rHuIL-12 delivered one day after TBI can significantly increase survival and reduce radiation-induced hematopoietic toxicity and infections. These data significantly advance development of rHuIL-12 toward approval under the Animal Rule as an effective stand-alone medical countermeasure against the lethal effects of radiation exposure.

## Introduction

Acute radiation syndrome (ARS) is a life-threatening illness caused by whole body or significant partial-body exposure to radiation doses > 1 Gy over a short period of time, as would occur in the event of a nuclear accident or attack [1,2]. The pathophysiology of ARS is well understood, and is similar across all mammals, involving detrimental effects on the hematopoietic, gastrointestinal, central nervous and cutaneous systems [3]. In the hematopoietic subsyndrome of ARS (HSARS), toxicity is due to rapid bone marrow ablation [1,2], leading to pancytopenia. HSARS ultimately results in death due to infection and/or hemorrhage over the range of 2 weeks to 2 months, depending on the radiation exposure level [1,2]. While the availability of a radiation medical countermeasure (R-MCM) in the event of a large scale radiation emergency is critical for saving lives, currently no treatments are approved as R-MCMs by the US Food and Drug Administration (FDA). Since early 1990s, preclinical studies have demonstrated radioprotective and/or radiomitigating properties of various cytokines and cytokine cocktails, but their further clinical development was hindered by adverse reactions [3-5]. A cocktail containing stem cell factor, FMS-like tyrosine kinase 3 ligand, thrombopoietin, and interleukin-3 with or without a long-acting pegylated form of granulocyte-colony stimulating factor (G-CSF) has been shown to improve survival in murine and primate models of acute radiation [6]. However, compared to the single agent approach, multicytokine combinations impose significant difficulties from the drug development perspective as well as logistical challenges for administration in the mass casualty scenario.

Typical treatment guidelines for HSARS include short- or long-term cytokine administration, depending on the radiation exposure level [3]. While available cytokine products support the growth of some individual cell types (such as G-CSF for neutrophils) [1], they do not consistently reduce overall mortality after TBI [7]. An optimal R-MCM against HSARS would be able to regenerate all blood cell lineages, and, given the expected logistic impediments of a mass casualty scenario, should be effective when administered hours to days after exposure, preferably as a single dose, and in the absence of intensive supportive care. These requirements are not fulfilled by G-CSF, which affects only the granulopoietic lineage, requires multiple daily administrations, and improves survival only in combination with intensive, trigger-based medical management [8]. Additionally, use of G-CSF in the context of radiation exposure was associated with delayed adverse effects, such as long-standing isolated thrombocytopenia [9] and lung toxicity [10].

We previously reported that a single administration of recombinant human IL-12 (rHuIL-12) given 24–25 hours after irradiation, in the absence of antibiotics, fluids or blood products, improved survival in both a murine HSARS model and in a proof-of-concept, open-label, male-only study in non-human primates (NHP) [11]. These findings supported the further development of rHuIL-12 as an R-MCM for HSARS under the FDA Animal Rule, where efficacy is proven in an appropriate animal model (eg, non-human primates [NHP]) and safety is demonstrated in humans. Herein, we describe results of a randomized, blinded, efficacy study of rHuIL-12 as an R-MCM in a larger group of male and female rhesus monkeys performed under Good Laboratory Practice (GLP). This study advances rHuIL-12 towards approval under the Animal Rule.

## Results

### Survival

**Figure 1:**
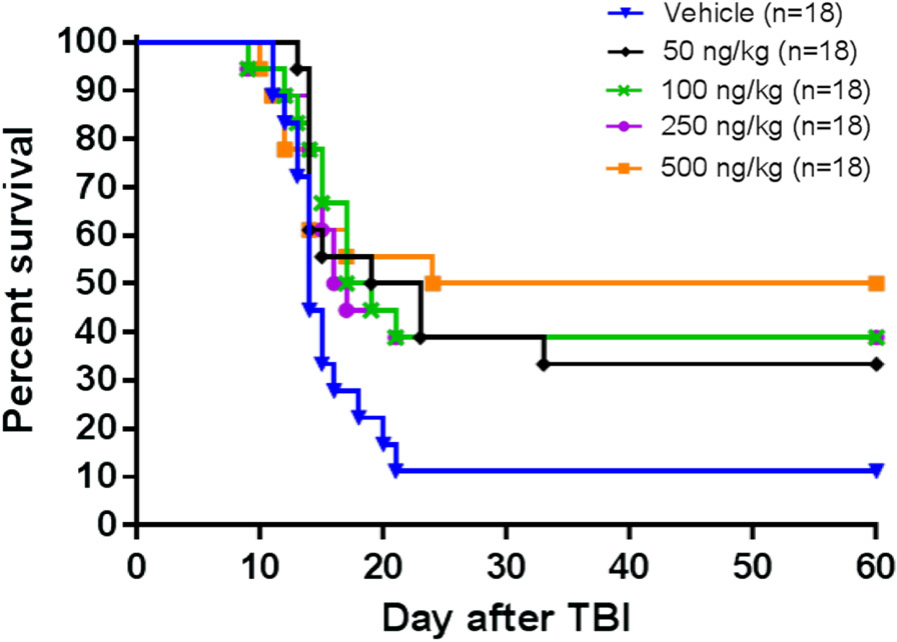
**Survival of rhesus monkeys following exposure to TBI and treatment 24 hours after TBI with either vehicle or rHuIL-12.** Kaplan-Meier plots of survival over the study period are shown for each treatment group. Each dose group comprised 18 animals. Log rank p-values were 0.0305 0.0344, 0.0404, and 0.0265, respectively for the 50 ng/kg, 100 ng/kg, 250 ng/kg and 500 ng/kg dose groups vs. the vehicle-treated control group.

Survival data are present in Figure 1. The administered radiation dose corresponded to an approximate LD_90/60_ (2/18 animals in the control groups) under the conditions of this experiment (no antibiotics, fluids or blood transfusions). Of the 59 deaths that occurred among rHuIL-12-treated animals, 58 occurred between Day 9 and Day 24, which is consistent with previously observed rates and timing of death due to HSARS in rhesus monkeys (CiToxLAB historical data), and one death occurred at Day 33. The highest proportion of deaths occurred between Day 11 and Day 21, with Day 14 being the peak day of death for both control and rHuIL-12-treated groups. The death rate was similar for males and females. All deaths, regardless of cause, were included in the statistical analysis of survival, the primary efficacy endpoint. As noted above, in the control group, 2 of 18 animals survived (11%, both males), while 33% (3 males and 3 females), 39% (4 males and 3 females), 39% (4 males and 3 females), and 50% (5 males and 4 females) of animals survived in rHuIL-12 treated groups 2–5, respectively. Each rHuIL-12 treated group showed a statistically significant increase in survival compared with the control group (log rank test p < 0.05). Pair-wise comparison between rHuIL-12-treated groups showed no significant differences. Possible causes of unscheduled death prior to day 60 were predominantly infection and hemorrhage (Table 1).

**Table 1:** Causes of death at unscheduled euthanasia

	**rHuIL-12 dose group (ng/kg)**					**All**
**Cause of death**	**0 (N = 16)**	**50 (N = 12)**	**100 (N = 11)**	**250 (N = 11)**	**500 (N = 9)**	**(N = 59)**
	**n (%)**	**n (%)**	**n (%)**	**n (%)**	**n (%)**	
Infection	13 (81.25)	12 (100)	10 (90.9)	10 (90.9)	8 (88.9)	53 (89.8)
Infection + hemorrhage	3 (18.75)	0	1 (9.1)	1 (9.1)	1 (11.1)	6 (10.2)

### Prodromal and clinical (manifest illness) observations

Vomiting and diarrhea occurred one or more times over the course of the study in more than half of all animals, with a higher incidence in the first few days following radiation. Incidence of vomiting and diarrhea was similar between the groups (Additional file 1: Table S1). Decreased activity was observed primarily between Days 4 and 24, with the largest decrease in the control group and smallest decrease in the group treated with highest rHuIL-12 dose (500 ng/kg; Additional file 1: Figure S1). Intermediate rHuIL-12 doses resulted in intermediate degrees of decreased activity, suggesting a possible inverse trend with rHuIL-12 dose. Decreased appetite was observed in two waves (Additional file 1: Figure S2): the first, on Days 1 through 4, occurred as an immediate reaction to irradiation; the second, on Days 10 through 34, paralleled the period of highest rates of infection, hemorrhage and death, as described below. During the first wave, the degree of decrease in appetite was similar between the control and rHuIL-12-treated groups, while during the second wave, a trend toward positive effect of rHuIL-12 was observed. Average body weight decreased by 5 to 10% from baseline beginning 3 to 4 days after irradiation and continuing up to Day 30, followed by recovery and subsequent increases above baseline (Additional file 1: Figure S3). The decrease in body weight was similar in the control and rHuIL-12-treated groups.

### Hematopoiesis

**Figure 2:**
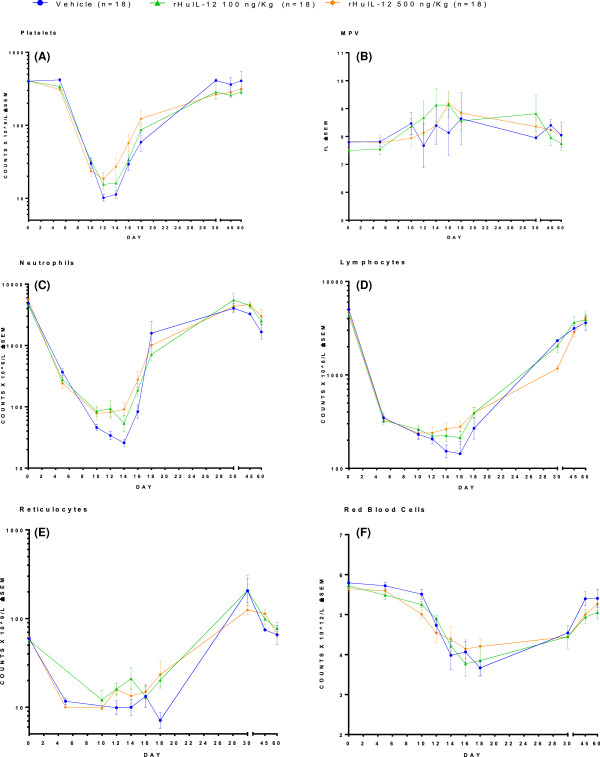
**Blood counts over time in rhesus monkeys exposed to lethal TBI and treated 24 hours after TBI with either vehicle or rHuIL-12 (Average ± SEM). A)** platelets; **B)** mean platelet volume; **C)** neutrophils; **D)** lymphocytes; **E)** reticulocytes; **F)** red blood cells. Normal ranges are as follows: platelets, 252 to 612 × 10^9^/L; mean platelet volume, 6.3 to 9.4 × 10^9^/L; neutrophils, 1.21 to 10.29 × 10^9^/L; lymphocytes, 1.85 to 8.71 × 10^9^/L; reticulocytes, 29.9 to 103.9 × 10^9^/L; red blood cells, 4.95 to 6.94 × 10^12^/L.

Blood counts for platelets, mean platelet volume, neutrophils, lymphocytes, and reticulocytes are presented in Figure 2 A–E, respectively. Hematology blood samples obtained at a pre-radiation time points were considered to be baseline levels. Blood samples obtained at post-radiation time points of 5, 10, 12, 14, 16 and 18 days corresponded to the period of severe cytopenias seen in the HSARS model. Additional samples obtained at days 30, 45 and 60 were used to evaluate whether levels returned to baseline in surviving animals.

#### Platelets

Platelet nadirs occurred at day 12 or 14, depending on the dosing group. Significant thrombocytopenia (<50 × 10^9^ /L) was present over days 10–15. The average platelet nadir in the control group (10.1 × 10^9^ platelets/L) was lower than that for each of the treated groups (12.1, 15.5, 12.7, and 18.6 × 10^9^ platelets/L for groups 2–5, respectively). By day 18, initial recovery was observed among survivors in all groups, with full recovery observed by day 30. The proportions of blood samples with severe thrombocytopenia (platelets <10 × 10^9^ /L) between Day 10 and 18 were 33% in the control group and 34%, 20%, 22% and 12% in rHuIL-12 treated groups 2–5, respectively. The pair-wise comparison of the proportion of blood samples with severe thrombocytopenia for rHuIL-12-treated animals and controls was significant for the 500 ng/kg group (Fisher exact test p = 0.0073). Mean platelet volume was increased between day 14 and 18, likely due to the release of young platelets from recovering bone marrow. Average peak values were 8.64 fL in the control group compared with 9.21, 9.13, 9.56 and 9.17 fL in rHuIL-12 treated groups 2–5, respectively.

#### Neutrophils

Neutrophil nadirs occurred between days 10 and 14, depending on dosing group. Severe neutropenia (<50 × 10^6^/L) occurred in 100% of animals in the control group and in 88.9%, 77.8%, 83.3%, and 72.2% of animals in rHuIL-12-treated groups 2–5, respectively. The average neutrophil nadir of the control group (26 × 10^6^ /L) was lower than that for each of the treated groups (34, 54, 39, and 78 × 10^6^/L in groups 2–5, respectively. Neutrophil recovery began by day 18 and baseline levels were reached by day 30. The percentage of blood samples presenting with severe neutropenia on days 10 to 18 was 67% in the control group compared to 46%, 35%, 46% and 31% in groups 2–5, respectively (Fisher’s exact test p = 0.0196, 0.0005, 0.0278, and < .0001, for comparison of control and groups 2–5, respectively).

#### Lymphocytes

Lymphocyte nadirs occurred between days 10 and 16, depending on dosing group. All groups showed severe lymphopenia down to 7 – 10% of pre-radiation levels. The average lymphocytes nadir in the control group (0.143 × 10^9^ /L) was lower than that for each of the treated groups (0.163, 0.213, 0.220, 0.239 × 10^9^/L for groups 2–5, respectively). On Day 18, initial recovery from the nadir was observed in all groups. By day 30, group average levels ranged from 30% to near 60% of the pre-radiation levels, and by days 45 and 60, counts were in the normal range but remained slightly lower than baseline levels. The difference in the lymphocyte nadir between controls and animals treated with rHuIL-12 was statistically significant in females treated at the 50, 250, and 500 ng/kg dose levels (Sidak adjusted t-test p = 0.0443, 0.0103, and 0.0211, respectively).

#### Red blood cells and reticulocytes

The red blood cells nadir occurred on Days 16–18, and represented a 37% reduction from baseline. Red blood cells nadirs were comparable in all groups (data not shown). Average reticulocyte nadirs were 7.1 × 10^9^/L in the control group and 8.7 × 10^9^/L, 12.1 × 10^9^/L, 9.1 × 10^9^/L, and 9.8 × 10^9^/L in rHuIL-12-treated groups 2–5, respectively, suggesting a stimulatory effect of rHuIL-12 on erythropoiesis. However, the differences did not reach statistical significance.

### Relationships between hematopoietic recovery and survival

Mean lymphocyte, platelet, neutrophil, and reticulocyte counts were higher in survivors than in decedents among the rHuIL-12 treated animals (in Additional file 1: Figure S4). Average platelet, neutrophil, and reticulocyte counts were higher in the survivors treated with rHuIL-12- than in survivors treated with vehicle control (in Additional file 1: Figure S4).

### Febrile neutropenia

A total of 15 animals (8 males; 7 females) had febrile neutropenia, and most (10/15) had been treated at the two highest dose levels (group 4, 250 ng/kg and group 5, 500 ng/kg) of rHuIL-12. Ten of the 15 animals with febrile neutropenia had a positive hemoculture; the most common bacteria identified were *Escherichia coli* and *Staphylococcus aureus*. Duration of febrile neutropenia was 1 day in all 15 affected animals and resulted in death on the same or next day in 12 of the 15 animals. Three of the 15 animals survived to Day 60 (1 in group 4 and 2 in group 5). Notably, 2 of the 3 surviving animals had negative blood cultures.

### Microbiology and pathology

#### Infection

In the control group hemoculture positivity was 86%, compared to 65%, 65%, 47% and 44% in rHuIL-12-treated groups 2–5, respectively. The difference was statistically significant for the two highest doses (Fisher’s exact test p = 0.0072 for each group). The decrease in the prevalence of infection was observed for both gram-negative and gram-positive bacteria. Bacteriological analysis of heart, kidney, liver, both lungs, brain and spleen performed at necropsy for all animals revealed a lower mean bacterial growth score in rHuIL-12-treated animals compared to that of controls (Table 2).

**Table 2:** Macroscopic organ hemorrhage score and organ infection score per animal (average ± SEM)

**Dose (ng/kg)**	**Total hemorrhage score**^**a**^	**GI tract hemorrhage score**^**b**^	**Total infection score**^**c**^
0	8.4 ± 1.6	5.6 ± 1.1.4	12.8 ± 2.2
50	5.1 ± 0.63	3.2 ± 0.75	12.3 ± 3.4
100	6.6 ± 0.73	4.5 ± 0.90	10.6 ± 3.7
250	5.0 ± 1.0	3.1 ± 0.78	8.8 ± 3.1
500	5.8 ± 1.1	3.7 ± 0.70	8.2 ± 3.3

^a^Hemorrhage was assessed on necropsy of found dead or preterminally euthanized animals by a pathologist blinded to the therapy group assignment. The following tissues were included in calculating the mean hemorrhage scores presented in this table: stomach, ileum, jejunum, duodenum, colon, cecum, rectum, heart, brain, kidneys, liver, lungs, urinary bladder). Hemorrhage score was defined as follows: 0 = absence of hemorrhage; 1 = minimal hemorrhage(s); 2 = slight hemorrhage(s); 3 = moderate hemorrhage(s); 4 = marked hemorrhage(s); and 5 = severe hemorrhage(s). In each animal scores for all organs were summed, then mean score for each treatment group was calculated.

^b^The following tissues were included in calculating the GI tract hemorrhage score: stomach, ileum, jejunum, duodenum, colon, cecum, and rectum.

^c^For infection score determination, organ samples were collected on necropsy and cultured (animals found dead excluded from the analysis), including brain, heart, kidney, liver, both lungs, and spleen. Bacterial growth was scored for each organ (from 0 to 4). In each animal scores for all organs were summed, then mean score for each treatment group was calculated.

*Escherichia coli* and *Staphylococcus aureus* were the most frequent isolates from organs and hemoculture. Among animals that underwent unscheduled euthanasia, 12 of 16 (75%) control animals had organ cultures that were positive for *Escherichia coli* compared with 66.7%, 63.6%, 72.7% and 55.6% of animals in rHuIL-12 treated groups 2–5, respectively. Similarly, 10 of the 16 (62.5%) control animals that underwent unscheduled euthanasia had organ cultures that were positive for *Staphylococcus aureus* compared with 50.0%, 54.5%, 54.5% and 44.4% of animals in rHuIL-12 treated groups 2–5, respectively.

### Pathology

#### Macroscopic evaluation of hemorrhage

Overall group mean hemorrhage scores for all organs, as well as a separate score for the gastrointestinal system, are shown in Table 2. Although the mean scores were higher in the control group than in all rHuIL-12-treated groups, the differences did not reach statistical significance, likely due to substantial organ to organ and animal to animal variation. Notably, the proportions of animals that had hemorrhage scores ≥ 4 in at least one organ were higher in the control group than in the groups treated with rHuIL-12, and brain hemorrhage was found only in 2 animals in the control group.

#### Microscopic evaluation

A wide range of microscopic findings were observed in numerous organs/tissues from monkeys who died or were euthanized before day 60. Microscopically, TBI-related hemorrhage was noted in numerous organs. Other microscopic findings related to TBI were noted in the bone marrow, lymphoid tissue, gastrointestinal tract, and kidney. Microscopic changes in many organs including small and large intestines, heart, liver, lungs, mesenteric lymph node, and spleen were considered to be predominantly secondary to episodes of bacteremia/septicemia. In animals surviving to day 60, microscopic findings related to irradiation were noted in lymphoid tissue, gastrointestinal tract, kidney, and bone marrow.

Except the hemorrhage reduction trend in the rHuIL-12 treated animals, there were no substantial differences between the characteristics or the incidence and/or severity of the macroscopic and microscopic findings in the control group compared to the rHuIL-12-treated groups, among both pre-terminally euthanized animals, animals that died, and surviving animals euthanized on day 60.

### Pharmacokinetics, pharmacodynamics and bone marrow assessment

In a companion study, a separate cohort of animals (2 per gender per group) was exposed to the same radiation level as in the survival study and treated with the same dose levels of rHuIL-12. Blood samples were collected at sequential time points from baseline through day 11 and analyzed for rHuIL-12, IFN-γ, IL-18, and IP-10. A dose dependent increase in rHuIL-12 exposure as well as increased plasma levels of IFN-γ, IL-18, and IP-10 were observed (Table 3).

**Table 3:** **C**_**max **_**and AUC**_**0-t **_**of rHuIL-12, IFN-γ, IL-18, and IP-10 after a Single Dose of rHuIL-12 in Irradiated Rhesus Monkeys**

	**rHuIL-12**		**IFN-γ**		**IL-18**		**IP-10**	
**Dose (ng/kg)**	**C**_**max **_**(pg/mL)**	**AUC**_**0-t **_**(hr · pg/mL)**	**C**_**max **_**(pg/mL)**	**AUC**_**0-t **_**(hr · pg/mL)**	**C**_**max **_**(pg/mL)**	**AUC**_**0-t **_**(hr · pg/mL)**	**C**_**max **_**(pg/mL)**	**AUC**_**0-t **_**(hr · pg/mL)**
0	BLQ	BLQ	150.20 (NR)	24868.80 (NR)	1920.75 (1207.67)	228818.77 (121013.28)	284.36 (188.72)	16519.02 (7951.53)
50	14.65 (4.75)	190.61 (90.51)	257.73 (120.42)	6239.20 (5395.40)	1831.76 (1275.11)	236796.45 (209796.17)	1100.67 (316.73)	43932.87 (29769.36)
100	39.03 (15.85)	532.72 (238.16)	709.60 (422.55)	22904.40 (8449.72)	2362.16 (1028.21)	309524.07 (123378.16)	1422.17 (120.53)	61099.10 (6735.34)
250	69.49 (11.47)	1397.30 (203.75)	1949.15 (1200.24)	76621.50 (41016.18)	3037.33 (1339.84)	391945.00 (150071.03))	2313.90 (1289.69)	78442.31 (17024.71)
500	137.18 (38.07)	2992.78 (1307.55)	3094.00 (1195.79)	123995.40 (42927.08)	4738.03 (1511.00)	621328.60 (171280.79)	3236.52 (3060.21)	118595.07 (57862.00)

All data are means (standard deviations).

*Abbreviations*: BLQ-Below the limit of quantitation; AUC, area under the concentration vs. time curve; 0-t, time 0 to time of assessment; hr, hour; NR, not reported due to < 3 animals contributing data.

**Figure 3:**
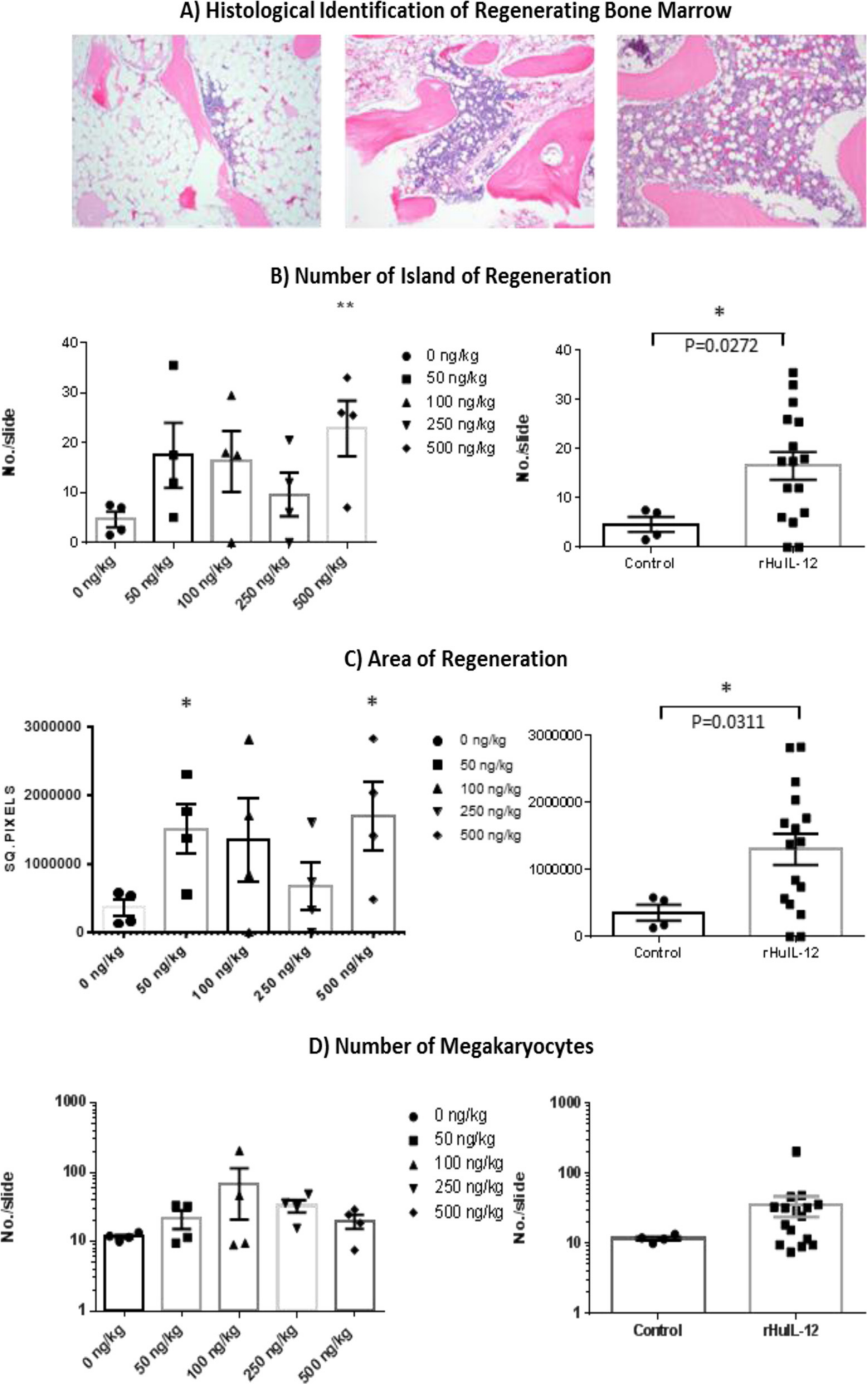
**Identification of Bone Marrow Regeneration Islands. (A)** Histopathological identification of regenerating bone marrow. Clusters of cells appearing in otherwise ablated bone marrow were scored as one regenerating island. Left panel, ablated bone marrow; middle panel, regenerating bone marrow; right panel, non-irradiated bone marrow. (Olympus BX41 compound microscope; Infinity Analyze software v5.0, magnification: 10×). **(B)** Quantification of number of islands of regeneration for individual treatment groups (left panel, p < 0.01 for 500 ng/kg group vs. control) and the combined rHUIL-12-treated groups vs. vehicle-treated control (right panel, p < 0.05). **(C)** Quantification of area of regeneration for individual treatment groups (left panel, p < 0.05 for 50 and 500 ng/kg groups vs. control) and the combined rHuIL-12-treated groups vs. vehicle-treated control (right panel, p < 0.05). **(D)** Quantification of megakaryocytes for individual treatment groups (left panel) and the combined rHuIL-12-treated groups vs. vehicle-treated control (right panel).

All animals were sacrificed at Day 12, which represented the day of estimated maximal bone marrow suppression based on the timing of nadir of blood cell counts in the previous study. Histological analysis of bone marrow showed severe hypocellularity with pockets of regeneration (Figure 3A). Quantitation of the number of regeneration islands, the total area of the regenerating islands, and the number of megakaryocytes in each animal was performed in a blinded analysis. Both the number and the area of the regenerative islands were higher in the rHuIL-12-treated groups compared with control, and the increases were similar across the rHuIL-12-treated groups (Figures 3 B-C). The difference for both parameters reached statistical significance for animal in group 5 (t-test p <0.01 and p <0.05 for number and area of islands of regeneration, respectively), as well as in a pooled comparison (all treated groups versus control group; p = 0.0272 and p = 0.0311 for number and area of islands of regeneration, respectively). Importantly, the number of megakaryocytes was also higher for all rHuIL-12­ treated groups relative to control, but the differences were not statistically significant (Figures 3D).

## Discussion

The data from this randomized, blinded, placebo-controlled study demonstrate a positive and significant effect of a single, subcutaneous injection of rHuIL-12, over a 10-fold dose range, on survival following lethal TBI (700 cGy; LD_90/60_) in the rhesus monkey model of HSARS. The animal model used in this study has been validated at CiToxLAB North America as an established model of human HSARS, based on the occurrence of similar hematologic effects, infection and hemorrhage following TBI as reported for humans [8,11]. Compared with other studies using a similar NHP model [8], our study did not include supportive care in the form of antibiotics, blood products and intravenous fluids, and therefore allows for a clearer demonstration of the effects of mitigating treatments and affords greater relevance for the mass casualty scenarios following a nuclear attack or accident, where intensive, hospital-based care would be significantly delayed and/or limited.

The current study, conducted under the FDA Animal Rule, provides a more robust demonstration of efficacy than our previously published proof-of-concept study [11], as it was blinded, randomized, and GLP compliant, and involved more than twice the number of animals.

The mechanism by which IL-12 rescues animals following TBI involves the multiple effects of IL-12 on hematopoieses and immune function. Radiation-induced bone marrow suppression was mitigated by rHuIL-12: animals treated with rHuIL-12 showed statistically significant reductions in the occurrence of severe neutropenia and severe thrombocytopenia, as well as attenuated nadirs for lymphocytes, neutrophils, platelets, and reticulocytes. Further, the increase, relative to controls, in mean platelet volume among animals treated with rHuIL-12 suggests that rHuIL-12 promoted release of newly formed platelets from the bone marrow. Quantitative analysis of the number and size of bone marrow regenerative pockets supports the conclusion that rHuIL-12 alone stimulates hematopoiesis, allowing for recovery of all major blood cell components.

Our in vivo observation that rHuIL-12 induced recovery of multiple hematopoietic lineages is consistent with previous reports in which IL-12 stimulated growth of hematopoietic stem cells and progenitors in vitro [12-14], and prevented radiation-induced death of hematopoietic stem cells in vivo in a murine model of HSARS [15]. This multilineage hematopoietic effect is also consistent with our previous findings in studies of tumor-bearing mice [16] and with our observation that IL-12 receptors are present on hematopoietic stem cells [11]. The observations that rHuIL-12 increased proportion of animals with higher blood counts, and higher blood counts were associated with survival, support the conclusion that rHuIL-12 improved survival by mediating early regeneration of multilineage bone marrow hematopoiesis, as compared to control.

Decrease of lymphocyte counts below 0.25 × 10^9^/L has been established as a marker of irreparable lethal bone marrow damage in a large database of human victims of acute radiation [17]. As such, it is important to note that in our study, the average lymphocyte nadir was 0.09 × 10^9^/L among decedents in the control group, 0.14 × 10^9^/L among decedents in the rHu-IL12-treated groups, and 0.27 × 10^9^/L among survivors in all groups. These findings further support the validity of our animal model as an accurate representation of human HSARS and its ability to predict effectiveness in humans exposed to lethal radiation.

Consistent with the reduction in severe neutropenia and lymphopenia, the incidence of blood culture positivity for infection was significantly lower in rHuIL-12-treated groups 4 and 5 (47% and 44%, respectively) compared with than in the control group (86%). These data demonstrate that rHuIL-12, administered 24 hours after TBI, in the absence of antibiotics, decreased infectivity of broad-spectrum bacteria. These effects are consistent with and are likely due to the well-known multiple stimulatory effects of IL-12 on innate and adaptive immunity [18]. On critical days 14–18 associated with deadly infections in this HSARS NHP model, the average lymphocyte counts in rHuIL-12 treated groups were higher than in control animals. The improvement of lymphocyte counts in this critical period may have provided enhancement of the T-cell-mediated immunity and B-cell antibody-mediated immunity (B cells) defense system, thereby contributing to higher survival in the treated groups as compared to the placebo group. Previous studies in mice have shown that during the early stages following exposure to lethal radiation, type 1 T-helper cell (Th1) function is reduced due to the suppression of endogenous IL-12 secretion from antigen presenting cells [19,20]. IL-12 administration may alleviate the radiation-induced impairment of Th1 function by promoting proliferation and activation of the NK cells, macrophages, and dendritic cells [21], which can be damaged by radiation [22]. Here we demonstrated that in irradiated monkeys rHuIL-12 increases plasma levels of IFN-γ, the hallmark of NK cell activation, as well as IL-18, and IP-10, similarly as we observed previously in non-irradiated monkeys [11]. The tri-directional cross-talk between NK, macrophages and dendritic cells further promotes their maturation [23], leading to the restoration of Th1 function and the establishment of early immune competence following TBI [11]. Further, continuous production of endogenous IL-12 from pathogen-activated dendritic cells serves as a positive feedback loop and plays a key role in sustaining the initial response to exogenous IL-12 [24]. Taken together, these IL-12 generated immune-mediated effects can account in large part for the positive survival benefit observed in this study.

Consistent with the reduction in severe thrombocytopenia, rHuIL-12 treatment in this study was associated with lower severity of hemorrhage for animals that died or were euthanized prior to the scheduled termination on Day 60. In support of our finding that treatment with rHuIL-12 was associated with reduced severe thrombocytopenia and hemorrhage, we recently reported that hematopoietic stem cells, megakaryocytes and osteoblasts in the bone marrow express the IL-12 receptor β2 subunit (IL-12Rβ2) [11], which is primary subunit for IL-12 signaling [25]. The presence of IL-12Rβ2 receptors on these key bone marrow cells suggest that through its receptors, rHuIL-12 may promote proliferation and differentiation of the surviving stem cells and megakaryocytes following exposure to lethal radiation, thereby enhancing platelets regeneration and reducing severe thrombocytopenia. Indeed, quantitative analysis of the bone marrow in our current companion study showed that relative to controls, rHuIL-12­ treated groups had higher numbers of megakaryocytes. The ability of rHuIL-12 to facilitate regeneration of platelets may be of clinical importance in indications other than HSARS mitigation, such as cancer, as there is currently no available drug that can facilitate platelet recovery following myelosuppressive therapies.

While leucocyte growth factors are recommended for use in victims of radiation, they are not approved by FDA for this indication. One published study of radiation mitigation in NHP demonstrated improved survival following exposure to lethal radiation for animals treated with rHuG-CSF in combination with intensive, trigger-based medical management (antibiotics, intravenous blood product transfusions, intravenous fluid replacement) compared with that of control animals that received only the medical management [8]. We recently completed a randomized, blinded study comparing a single injection of rHuIL-12 or vehicle with 18 injections of rHuG-CSF in the NHP model without supportive care. Survival analysis confirmed superior survival in the rHuIL-12-treated group vs. both the control group and a G-CSF-treated group. Notably, G-CSF did not provide any survival benefit compared to control (Gluzman-Poltorak et al., submitted for publication).

In parallel to the animal efficacy studies, the safety and tolerability of rHuIL-12 has been examined in normal healthy subjects per the Animal Rule. A first in human (FIH) study was conducted to determine the safe and well-tolerated doses of rHuIL-12 via dose escalation (at doses ranging from 2 to 20 μg). The FIH study was followed by a phase 1b expansion study at the highest safe and well-tolerated dose from the FIH study of 12 μg (Gokhale et al., accepted for publication). The 12 μg unit human dose for a 70 kg adult can be converted to 171 ng/kg rhesus monkey dose using a weight based conversion and this dose is within the efficacious dose range as determined in our rhesus monkeys studies.

## Conclusion

In summary, this randomized, placebo-controlled, blinded study has demonstrated that rHuIL-12 is an effective frontline radiomitigator due its ability to increase survival and regenerate the hematopoietic system when administered as a single, low dose without supportive antibiotics, fluids or blood products 24 hours following exposure to lethal radiation. Translation of the efficacious and safe dose from an animal model to humans is a significant challenge for any drug development program under the Animal Rule. Thus, our finding that statistically significant increases in survival can be achieved over a ten-fold effective dose range of rHuIL-12 in the NHP model will provide a distinct advantage for optimal human dose selection.

## Methods

### Ethics statement

All procedures were reviewed and approved by the Institutional Animal Care and Use Committee (IACUC) of CiToxLAB Research, Inc.

### Animals

Studies were conducted in compliance with the Good Laboratory Practice (21 CFR Part 58) at CitoxLAB North America (a Contract Research Organization, Montreal, Quebec, Canada). Rhesus monkeys (*Macaca mulatta*) were obtained from the Yongfu County Xingui Wild Animals Raising Ltd., China. Monkeys (3 to 5 years old, and 3.0 to 5.7 kg at the start of treatment) were housed individually and acclimated for ≥ 5 weeks prior to irradiation. Harlan Teklad Certified Hi-Fiber Primate Diet #7195C (Harlan Laboratories, Indianapolis, Indiana) was provided twice daily.

### Experiment design

The dose of 700 cGy (60 cGy/minute from a Theratron 1000 Co^60^ source [Best Theratronics; Ottawa, Ontario, Canada]) was based on available historical data from CiToxLAB North America. TBI was conducted with animals in a vertical position, as described previously [11]. For homogenous dose distribution, the first half-dose was delivered anteroposterior and the second half-dose was delivered posteroanterior. Dosimetry was verified to be within 10% of prescribed dose using nanodot chips (Landauer, Inc., Glenwood, Illinois, USA) positioned on the front and back of each animal.

In the main study, male and female animals (45 each; 9 animals per sex per dose group) were randomized, stratified by body weight to the following doses of clinical grade rHuIL-12 administered by SC injection between the scapulas approximately 24–25 hours following TBI: vehicle control (Group 1), 50 ng/kg (Group 2), 100 ng/kg (Group 3), 250 ng/kg (Group 4), or 500 ng/kg (Group 5). The concentration of the test item in each dosing sample was verified by Intertek Pharmaceutical Services (San Diego, CA) using the Quantikine® Human IL-12 ELISA Kit (R&D Systems Inc., Minneapolis, USA). The pathologist and study staff other than the study team leader and those involved with irradiation were blinded. Specifically, animal care and euthanasia decisions were made by the blinded personnel.

### Symptomatic care

The following products were permitted as symptomatic care: buprenorphine (0.01-0.015 mg/kg/dose BID or TID, SC) for pain; bupivacaine (0.25%) topically for management of mouth ulcers; Pepto-Bismol for management of diarrhea; snacks or supplements (Rhesus Liquid Diet [BIO-SERV; Frenchtown, New Jersey], Ensure® [Abbott Laboratories, Abbott Park, Illinois, USA], vegetables, juices, or crushed cookies with banana) for anorexia; topical hydrotherapy and/or iodine 1% for wounds.

### Assessments

Decreases in appetite (based on food intake) and physical activity were recorded daily and scored as follows: 1 = slight; 2 = moderate; and 3 = severe. A detailed physical examination was performed prior to rHuIL-12 dosing and twice weekly thereafter. Body temperature (auricular) was taken prior to irradiation and on Days 3–10, 12, 14, 16, 18, 30, 45, and 60, or when clinically justified. Blood sampling (0.5 mL) for peripheral blood counts was performed prior to irradiation and at Days 5, 10, 12, 14, 16, 18, 30, 45, and 60. Blood was collected for hemoculture in cases of febrile neutropenia (absolute neutrophil count <0.05 10^9^/L together with rectal body temperature ≥104° F/40.0°C) and at necropsy.

### Terminal procedure

Animals were euthanized prior to Day 60 if any of the following criteria were observed: respiratory distress; complete anorexia for 3 day; loss of > 20% of initial body weight over a 3 day period; severely decreased activity level (recumbent during an entire observation period or unresponsiveness to touch); acute loss of > 20% estimated blood volume; generalized seizure activity; abnormal appearance (posture, rough coat, head down, exudates around eyes and nose, pallor, tucked abdomen and clinical appearance) associated with abnormal vital signs: severe dehydration with hypothermia (decreasing rectal temperature reaching <34.6°C and severely decreased activity level) or hyperthermia (temperature >40.1°C and severely decreased activity level). Euthanasia decisions were made by a team of technicians and veterinarians blinded to the animal group assignment. Surviving animals were euthanized at Day 60 following TBI.

Necropsy comprised an external macroscopic examination, a detailed internal examination, evaluation of organ weights and gross pathology, and collection of tissues for histopathology. Presence of hemorrhage was scored for major organs as follows: 0 = absence; 1 = minimal; 2 = slight; 3 = moderate; 4 = marked; 5 = severe. For histological examination, tissues were embedded in paraffin, sectioned and stained with hematoxylin and eosin-phloxin (H & E).

Microbiological analysis was conducted on brain, heart, kidney, liver, both lungs, and spleen. Bacterial growth was scored (0 to 4) for each organ. The total score was summed for each animal; the mean score was calculated for each treatment group.

### Pharmacokinetics and pharmacodynamics of rHuIL-12 and bone marrow histopathology in irradiated rhesus monkeys

An evaluation of pharmacokinetics and pharmacodynamics of rHuIL-12 in irradiated rhesus monkeys was conducted using separate animals randomized to the same doses of rHuIL-12 as in the survival cohort (2 per sex per group). Blood samples from animals treated with SC rHuIL-12 or placebo were collected at the following time points: pretreatment (approximately 2 weeks prior to irradiation), 24 hours after irradiation immediately before rHuIL-12 dosing, and at 1, 3, 5, 8, 12, 24, 48, 72, 96, 120, 144, 240, and 264 hours after rHuIL-12 dosing. The concentrations of rHuIL-12 and IFN- γ in monkey plasma were determined by validated GLP ELISA methods at Intertek ALTA Analytical Laboratory (San Diego, CA). rHuIL-12 was measured using the Human IL-12 HS ELISA kit (catalog # PHS120, HS120, or SS120 or equivalent; R&D Systems). The lower limit of quantitation was 3.5 pg/mL in 100% monkey plasma. IFN-γ was measured using the Monkey IFN-γ ELISA kit (catalog # 3420 M-1H-20, or equivalent; Mabtech, Inc., Mariemont, OH ). The lower limit of quantitation was 7.5 pg/mL in 100% monkey plasma. Interleukin-18 (IL-18) and interferon γ-induced protein (IP-10) levels were determined using non-GLP qualified ELISA methods at Neumedicines, Inc. IL-18 was assayed using the MBL International Corporation Human IL-18 ELISA (R&D Systems, Minneapolis, MN). The lower limit of quantitation was 120 pg/mL in 100% monkey plasma. IP-10 concentrations were determined in plasma using a Quantikine Human CXCL10/IP-10 ELISA (R&D Systems, Minneapolis, MN). The lower limit of quantitation was 15 pg/mL in 100% monkey plasma. Standard non-compartmental analyses were performed using Phoenix™ WinNonlin® Version 6.3 (WinNonlin; Pharsight Corporation, Mountain View, CA).

The separate cohort animals were euthanized on Day 12 after TBI, except one animal that underwent unscheduled euthanasia on Day 11. All animals were included in the bone marrow analysis. Two H & E femur sections for each animal were scanned on an Olympus BX41 compound microscope. Images of approximately 40 fields of view encompassing each femur section in its entirety were acquired on Infinity Analyze software v5.0 at a magnification of 10×. The number of bone marrow regeneration islands was determined by visual quantification in each field of view in each section. The total area of bone marrow regeneration was determined using ImageJ software, version 1.46. The mean number of regeneration islands and mean area of regeneration from two sections per animal were used in the statistical analyses. The number of megakaryocytes was determined visually in each femur section.

### Statistical analysis

All statistical comparisons were conducted for sex and for the entire study population. Survival functions were estimated using the Kaplan-Meier product-limit method applied on daily intervals. The control group was compared to each of the other treated groups using the Mantel log-rank test. Predefined GLP survival analysis was performed at CiToxLAB.

Group comparisons for incidences of severe neutropenia (defined as neutrophil count < 0.05 × 10^9^/L), severe thrombocytopenia (defined as platelet count < 10 × 10^9^/L), and hemoculture positivity (sepsis) were performed using Fisher Exact test. If the overall comparison was significant (p ≤ 0.05), pair-wise comparisons between control group and each dosing group was done using the Fisher Exact test.

Group means for bone marrow regeneration data (number and area of regeneration islands) were compared by a one-tailed t­ test using the statistical software program Prism version 6 (GraphPad, San Diego, CA). Differences with p <0.05 were considered significant.

## Abbreviations

ARS: Acute radiation syndrome; FDA: US food and drug administration; GLP: Good laboratory practice; G-CSF: Granulocyte-colony stimulating factor; HSARS: Hematopoietic syndrome of the acute radiation syndrome; IL-12Rβ2: IL-12 receptor β2 subunit; NK: Natural killer; rHuIL-12: Recombinant human interleukin-12; R-MCM: Radiation medical countermeasure; Th1: T-helper cell type 1.

## Competing interests

All authors are employees of and own equity of $10,000 or more in Neumedicines, Inc. The authors declare that they have no competing interests.

## Authors’ contributions

ZGP designed research, performed research, collected data, analyzed and interpreted data, performed statistical analysis, and wrote the manuscript. SRM performed research, collected, analyzed and interpreted data, and wrote the manuscript. VV analyzed and interpreted data, performed statistical analysis, and wrote the manuscript. HK collected and analyzed data. LAB designed research, analyzed and interpreted data, and wrote the manuscript. All authors read and approved the final manuscript.

## Supplementary Material

Additional file 1: Table 1 Percentage of animals presenting with selected early clinical signs on one or more days following TBI. **Figure 1.** Decrease in physical activity. **Figure 2.** Decrease in appetite score. **Figure 3.** Body weight over time. **Figure 4.** Blood counts over time in surviving vs. non-surviving rhesus monkeys exposed to lethal TBI and treated 24 hours after TBI with either vehicle or rHuIL-12 (Average ± SEM). Click here for file
