# Supplementary material for: Randomized comparison of single dose of recombinant human IL-12 versus placebo for restoration of hematopoiesis and improved survival in rhesus monkeys exposed to lethal radiation
Source: J Hematol Oncol. 2014 Apr 6;7:31. doi: 10.1186/1756-8722-7-31 (PMC4108131; doi:10.1186/1756-8722-7-31)

**Supplemental Table and Figures**

**Supplemental Table 1. Percentage of Animals Presenting with Selected Early Clinical Signs on One or More Days Following TBI**

|  | **rHuIL-12 Dose Group (ng/kg)** | | | | |
| --- | --- | --- | --- | --- | --- |
| **Clinical Sign** | **0  (N= 18)** | **50  (N= 18)** | **100  (N= 18)** | **250  (N= 18)** | **500  (N= 18)** |
|  | **n (%)** | **n (%)** | **n (%)** | **n (%)** | **n (%)** |
| **Vomiting** | **13 (72.2)** | **12 (66.7)** | **9 (50)** | **11 (61.1)** | **13 (72.2)** |
| **Diarrhea** | **12 (66.7)** | **10 (55.6)** | **10 (55.6)** | **12 (66.7)** | **14 (77.8)** |

**Supplemental Figure 1. Decrease in Physical Activity.** Average score for decrease in activity, calculated per live animal, by dose groups. Scoring rubric: 1 = slight decrease; 2 = moderate decrease; 3 = severe decrease.


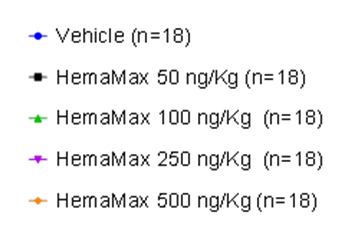
**
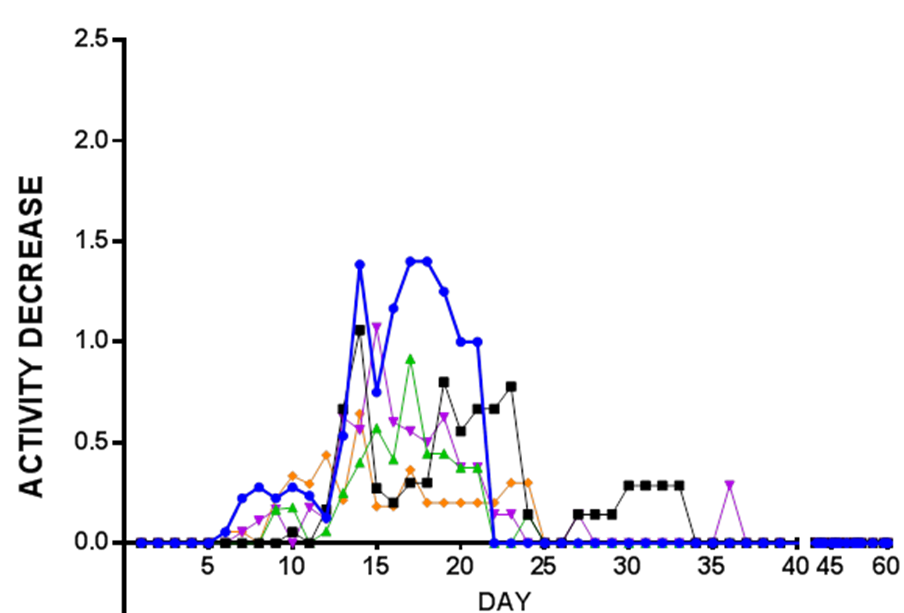
**


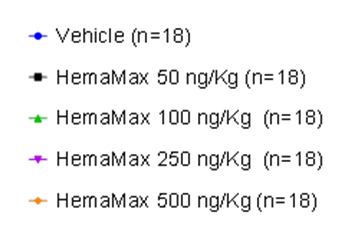
**
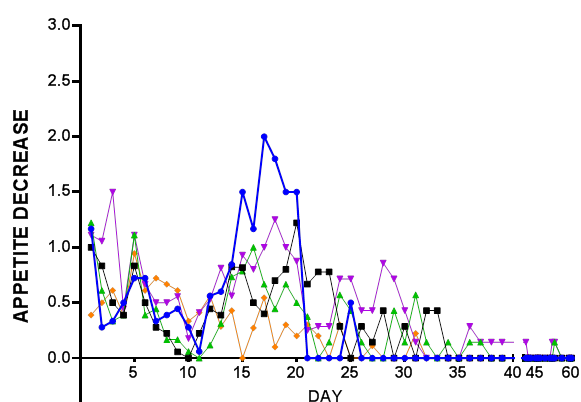
Supplemental Figure 2. Decrease in Appetite Score.** Average score for the decrease in appetite, calculated per live animal, by dose groups. Scoring rubric: 1 = slight decrease; 2 = moderate decrease; 3 = severe decrease.


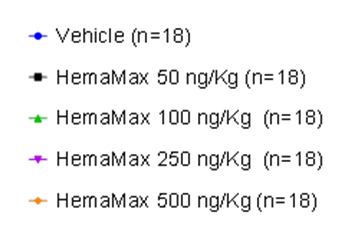
**Supplemental Figure 3. Body Weight over Time.** Average percent body weight (± standard error of the mean) relative to baseline body weight, by dose group, over time.

**Supplemental Figure 4. Blood counts over time in surviving vs. non-surviving rhesus monkeys exposed to lethal TBI and treated 24 hours after TBI with either vehicle or rHuIL-12 (Average ± SEM).**

A) lymphocytes; B) neutrophils; C) platelets; D) reticulocytes. Normal ranges are as follows: lymphocytes, 1.85 to 8.71 x 10^9^/L; neutrophils, 1.21 to 10.29 x 10^9^/L; platelets, 252 to 612 x 10^9^/L; reticulocytes, 29.9 to 103.9 x 10^9^/L.


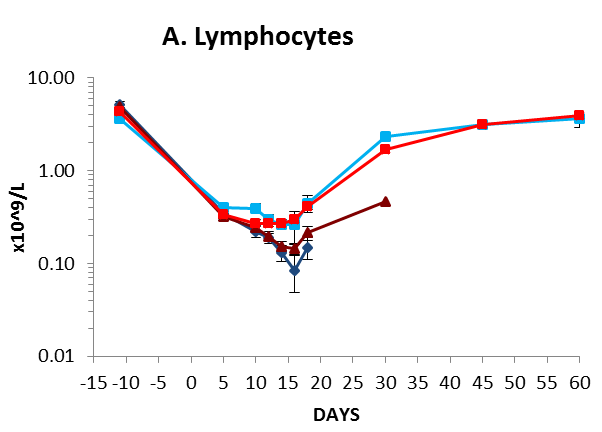

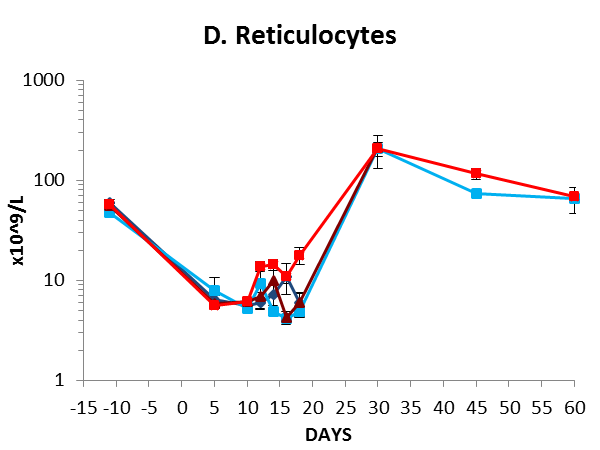

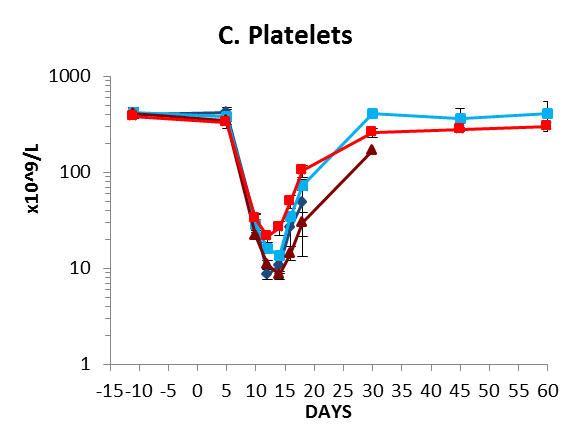

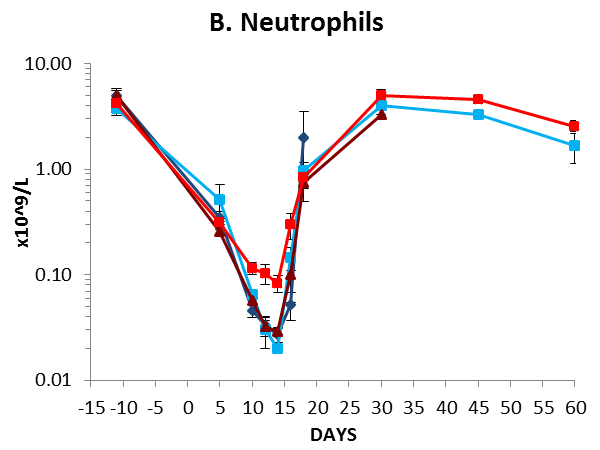

Supplement: Additional file 1: Table 1 — Percentage of animals presenting with selected early clinical signs on one or more days following TBI. Figure 1. Decrease in physical activity. Figure 2. Decrease in appetite score. Figure 3. Body weight over time. Figure 4. Blood counts over time in surviving vs. non-surviving rhesus monkeys exposed to lethal TBI and treated 24 hours after TBI with either vehicle or rHuIL-12 (Average ± SEM). [file 1756-8722-7-31-S1.docx]
